# Geometry-Tunable Nanoneedle Arrays Reveal Membrane Penetration Mechanics for Intracellular Delivery

**DOI:** 10.3390/bios16090495

**Published:** 2026-09-04

**Authors:** Xuanhe Zhang, Zheng Wang, Yiqing Chen, Lele Song, Yuan Ma, Jiadao Wang

**Affiliations:** 1 State Key Laboratory of Tribology in Advanced Equipment, Tsinghua University, Beijing 100084, China; 2 Key Laboratory for Micro/Nano Optoelectronic Devices of Ministry of Education & Hunan Provincial Key Laboratory of Low-Dimensional Structural Physics and Devices, School of Physics and Electronics, Hunan University, Changsha 410082, China

**Keywords:** geometry-tunable nanoneedle arrays, micro/nano fabrication, cell penetration mechanics, intracellular delivery, microfluidics

## Abstract

Nanoneedle arrays provide a promising interface for intracellular delivery, yet scalable control of array geometry and membrane penetration mechanics remains insufficiently understood. Here, we developed a rapid and scalable strategy for fabricating geometry-tunable silicon nanoneedle arrays. One-step SF_6_/O_2_ etching produced ordered arrays with center-to-center spacing of 1–5 μm, whereas pseudo-Bosch etching produced high-aspect-ratio (HAR) nanoneedles. Using a microwell-assisted cell-on-probe atomic force microscopy platform, we quantified the first penetration force, penetration probability, and number of penetration events at the single-cell level. For one-step-etching arrays, increasing spacing from 1 to 5 μm reduced the first penetration force from 34.87 ± 2.90 to 4.05 ± 0.30 nN and increased the penetration probability from 0.21 ± 0.03 to 0.87 ± 0.04. A phenomenological inverse-square model captured the force–spacing relationship, supporting an array-level load-sharing mechanism. Under identical vibration-assisted microfluidic conditions, the FITC-dextran-positive fraction increased from 22.8% for 1 μm arrays to 77.1% for 5 μm arrays, whereas 1 μm HAR arrays achieved 28.8%. These results identify array spacing as a key factor governing single-cell penetration and delivery and provide a mechanistic basis for nanoneedle-based biosensor and cell-interface design.

## 1. Introduction

Intracellular delivery serves as a fundamental step in cell therapy, regenerative medicine, tissue engineering, and biomanufacturing [1,2,3]. Efficient delivery of functional biomolecules is essential for these applications, yet the cell membrane acts as a highly selective biological barrier that restricts the direct entry of exogenous molecules [4]. Although liposome-based delivery and other chemical transfection methods can achieve high delivery efficiency, they often require specific optimization across different cell types and cargoes and may suffer from cytotoxicity or inconsistent performance. Similarly, viral transduction provides efficient gene transfer but raises manufacturing complexity and potential safety concerns such as uncontrolled genomic integration [5,6,7]. Polymeric nanocarriers have broadened nonviral gene delivery, but performance remains dependent on carrier chemistry and cell- and cargo-specific optimization [8]. In comparison, physical delivery strategies transiently disrupt the cell membrane by mechanical forces, therefore providing vector-free alternatives with broader applicability to diverse cell types and molecular cargoes. Among these approaches, nanoneedles have emerged as versatile micro/nanostructured interfaces for locally contacting, deforming, or penetrating cell membranes and have been increasingly used in developing intracellular delivery, cell engineering, and biosensing devices [3,9,10,11].

Despite the promising biomedical applications of nanoneedle arrays, scalable applications are still largely constrained by fabrication strategy [12,13,14,15]. Common approaches for fabricating silicon-based nanoneedles include chemical vapor deposition (CVD), reactive ion etching (RIE), metal-assisted chemical etching (MACE), atomic layer deposition (ALD), and anisotropic wet etching [3,9]. CVD enables epitaxial growth of nanowires and offers potential for industrial-scale production, but it typically requires metal catalysts, high-temperature growth, catalyst patterning, and complex process control [16,17,18]. MACE uses metal catalysts and HF/H_2_O_2_-based wet etching to achieve anisotropic silicon etching, but it also involves complex processing, potential metal residues, and environmental concerns associated with highly corrosive reagents [19,20,21]. ALD provides atomic-level thickness control, but its low deposition throughput and relatively high cost limit its applicability to high-throughput fabrication [22,23]. Anisotropic wet etching is simple and low-cost, yet the resulting structures are constrained by crystallographic planes, making it difficult to independently control nanoneedle morphology [24,25]. Inductively coupled plasma (ICP) etching or RIE combined with photolithographic masks can generate nanostructure arrays with high precision, but conventional photolithography-based methods generally require expensive exposure systems and multiple fabrication steps [26,27,28]. In contrast, self-assembled micro/nanosphere masks provide a promising route for low-cost, large-area nanoneedle fabrication. Nevertheless, precise control over key geometric parameters, including nanoneedle spacing, tip radius, and aspect ratio, remains a central challenge for this strategy.

Existing studies of nanoneedle arrays have mainly relied on imaging or modeling to evaluate array–cell interactions [29,30,31], whereas most AFM studies have quantified membrane penetration using a single nanoneedle and therefore describe an isolated tip–cell interaction [32,33,34]. Other array-based studies have evaluated intracellular delivery outcomes [35,36,37,38], but direct force measurements of simultaneous array–cell interactions remain limited. This gap restricts mechanistic interpretation of how array geometry affects membrane penetration and device performance. Beyond biomedical interfaces, tip effects in micro/nanostructures have also been explored for energy conversion, water capture, and environmental remediation [39]. Scalable development of nanoneedle-based devices also requires rapid, cost-effective fabrication of large-area arrays with tunable geometry. Here, we developed a self-assembly-assisted fabrication method compatible with 4-inch-wafer processing. One-step etching produced arrays with 1–5 μm spacing, while pseudo-Bosch etching produced HAR arrays. We further used a microwell-assisted cell-on-probe AFM platform to quantify the penetration mechanics between individual Jurkat E6-1 cells and nanoneedle arrays. A phenomenological inverse-square model was used to describe the force–spacing relationship during cell penetration, and the corresponding arrays were evaluated under fixed continuous-flow delivery conditions. Together, this work provides a rapid method for fabricating large-area nanoneedle arrays and clarifies how array geometry influences membrane penetration at the single-cell level, providing a mechanistic basis for the development of nanoneedle-based delivery and biosensing devices.

## 2. Materials and Methods

### 2.1. Cell Culture

The Jurkat E6-1 cell line was obtained from the Cell Resource Center of Peking Union Medical College, Beijing, China (1101HUM-PUMC000075). The Jurkat E6-1 cells were cultured in RPMI-1640 medium (Gibco, Thermo Fisher Scientific, Waltham, MA, USA, 61870036) supplemented with 10% fetal bovine serum (FBS, Gibco, A5670701) and 1% penicillin–streptomycin (Gibco, 15240-062). Cells were maintained in a humidified incubator (Thermo Fisher Scientific, 311) at 37 °C with 5% CO_2_. During routine culture, the cell density was maintained between 1 × 10^6^ and 2 × 10^6^ cells/mL. All experiments were conducted within 5 cell line passages.

### 2.2. Self-Assembly and Pattern Transfer of Polystyrene (PS) Microsphere Monolayers

A monolayer of PS microspheres was fabricated via a surface tension gradient–driven self-assembly method [40]. After ultrasonication, a PS microsphere suspension (diameter: 1–5 μm) was injected onto the surface of deionized water using a syringe pump at a flow rate of 100 μL/min, forming an initially loosely packed monolayer. Subsequently, a surfactant-loaded hydrogel was placed at the interface to generate a lateral surface tension gradient, which continuously compressed the microspheres into a close-packed monolayer. A silicon substrate was placed underneath the water surface and slowly lifted through the air-water interface using a micromotion stage. The close-packed PS microsphere monolayer was then transferred onto the silicon surface and dried under ambient conditions.

### 2.3. Fabrication of Geometry-Tunable Nanoneedle Arrays

4-inch silicon wafers patterned with close-packed PS microsphere monolayers were used as substrates for nanoneedle fabrication. Nanoneedle arrays were fabricated by ICP etching using a GSE-C200 system (NAURA Technology Group Co., Ltd., Beijing, China). For one-step etching, O_2_ plasma was first used to shrink the PS microspheres and tune the exposed silicon area. The O_2_ plasma shrinkage was performed under the following conditions: ICP power, 200 W; radio-frequency (RF) power, 20 W; chamber pressure, 25 mTorr; O_2_ flow rate, 40 sccm; and chamber temperature, 48 °C. The O_2_ etching times for PS microspheres with diameters of 1, 2, 3, 4, and 5 μm were 40, 60, 120, 160, and 200 s, respectively. Silicon etching was then performed using SF_6_/O_2_ plasma to form nanoneedle arrays. The silicon etching conditions were as follows: RF power, 200 W; chamber pressure, 25 mTorr; and chamber temperature, 48 °C. For PS microspheres with diameters of 1, 2, 3, 4, and 5 μm, the corresponding etching times and SF_6_/O_2_ flow rates were 180 s at 15/20 sccm, 300 s at 20/25 sccm, 460 s at 25/30 sccm, 500 s at 30/35 sccm, and 650 s at 35/40 sccm, respectively. Residual PS masks were removed by ultrasonic cleaning, and the wafers were diced into 2.5 cm × 2.5 cm pieces for subsequent experiments. For HAR nanoneedle fabrication, pseudo-Bosch etching was performed by alternating C_4_F_8_ passivation and SF_6_ etching steps. Each cycle consisted of a C_4_F_8_ deposition step followed by an SF_6_ etching step. The C_4_F_8_ deposition step was performed under the following conditions: ICP power, 500 W; RF power, 50 W; chamber pressure, 25 mTorr; C_4_F_8_ flow rate, 100 sccm; chamber temperature, 30 °C; and deposition time, 10 s. The subsequent SF_6_ etching step was performed under the following conditions: ICP power, 500 W; RF power, 50 W; chamber pressure, 25 mTorr; SF_6_ flow rate, 20 sccm; chamber temperature, 30 °C; and etching time, 8 s. The optimized HAR nanoneedle arrays were obtained after 100 cycles of pseudo-Bosch etching.

### 2.4. Fabrication of Microchannel Mold

Microchannel molds were fabricated using standard soft lithography based on negative photoresist patterning. SU-8 2010 (Kayaku Advanced Materials, Westborough, MA, USA) photoresist was spin-coated onto cleaned silicon wafers at 1000 rpm to form a uniform photoresist layer. The coated substrates were soft-baked at 65 °C for 5 min and subsequently at 95 °C for 10 min, followed by gradual cooling to room temperature. Microchannel patterns were designed using AutoCAD 2022 (Autodesk, San Francisco, CA, USA) and transferred onto the photoresist using a laser direct writing system (TuoTuo Technology (Suzhou) Co., Ltd., Suzhou, China, UV Litho-ACA). After exposure, post-exposure baking was conducted under the same conditions as the soft-bake. The exposed samples were developed in propylene glycol monomethyl ether acetate (PGMEA, Sigma-Aldrich, St. Louis, MO, USA, 484431), rinsed with isopropanol, and hard-baked at 150 °C for 5 min to obtain the microchannel mold.

### 2.5. Fabrication of Nanoneedle–Microfluidic Chip

Nanoneedle surface patterning was performed by standard photolithography. SU-8 2002 (Kayaku Advanced Materials) photoresist was spin-coated onto the nanoneedle substrate at 3000 rpm to define the bonding area. The coated samples were soft-baked at 65 °C for 5 min and 95 °C for 10 min, followed by cooling to room temperature. The pattern was designed using AutoCAD 2022 (Autodesk) and fabricated by laser direct writing (TuoTuo Technology, UV Litho-ACA). After post-exposure baking under the same conditions, the patterned structures were developed in PGMEA (Sigma-Aldrich, 484431) and hard-baked at 150 °C for 5 min. Polydimethylsiloxane (PDMS, Sylgard 184, Dow Corning, Midland, MI, USA) prepolymer and curing agent were mixed at a weight ratio of 10:1, degassed under vacuum, poured onto the microchannel mold, and cured at 80 °C for 1 h. The cured PDMS microchannel layer was peeled off, and inlet/outlet holes were punched. Prior to bonding, both the PDMS microchannel and the patterned nanoneedle substrate were treated with oxygen plasma for 60 s (50 W, 20 sccm) and immediately brought into contact to form irreversible bonding.

### 2.6. Characterization

The geometry of microchannels was examined using a metallographic microscope, and the microchannel height was quantified by three-dimensional white-light interferometry (ZYGO NexView, Middlefield, CT, USA). The morphology of nanoneedle arrays and microchannels was characterized by scanning electron microscopy (SEM; Gemini 450, Carl Zeiss Microscopy GmbH, Oberkochen, Germany). Energy-dispersive X-ray spectroscopy (EDS) was performed using an X-Max 80 detector (Oxford Instruments NanoAnalysis, High Wycombe, UK) integrated with Gemini 450, and the data were analyzed using AztecOne (Oxford Instruments NanoAnalysis). EDS characterization was performed at an accelerating voltage of 15 kV, a working distance of 8.5 mm, and an acquisition time of 2 min. Elemental composition was determined from the map sum spectrum. For nanoneedle observation, samples were mounted on a tilted stage at an angle of 45° to visualize the needle profiles. SEM imaging was performed at an accelerating voltage of 5 kV with an aperture size of 120 μm. The Dimension Icon AFM (Bruker, Santa Barbara, CA, USA) was used to characterize the surface topography of the nanoneedle array under tapping mode. The AFM data were analyzed using NanoScope Analysis v1.90.

Cell morphology and fluorescence signals were observed using a bright-field microscope (DSZ2000X; Chongqing UOP Photoelectric Technology Co., Ltd., Chongqing, China) and a fluorescence microscope (EVOS M7000; Thermo Fisher Scientific). Intracellular delivery efficiency and fluorescence intensity were quantitatively analyzed by flow cytometry (Attune NxT; Thermo Fisher Scientific).

### 2.7. Intracellular Delivery Procedure

Before delivery experiments, the microchannels were incubated with 0.1% Poloxamer F68 (Gibco, 24040032) for 1 h to reduce nonspecific cell adhesion, followed by rinsing with 3 mL phosphate-buffered saline (PBS; Gibco, 10010023). Cells were resuspended in PBS containing 10 μg/mL FITC–dextran (10 kDa; MedChemExpress, Monmouth Junction, NJ, USA, HY-128868), and the cell density was adjusted to 2 × 10^6^ cells/mL. The cell suspension was introduced into the nanoneedle-microfluidic chip using a syringe pump (Chemyx, Stafford, TX, USA, FUSION 100-X) at a constant flow rate of 200 μL/min. During continuous-flow delivery, vertical vibration was applied using a custom-built vibration stage with a sinusoidal waveform and an acceleration amplitude of 8 g. After delivery, the collected cells were incubated at 37 °C in the dark for 20 min. The cells were then washed three times with PBS and used for flow cytometry or fluorescence microscopy characterization. For post-delivery cell culture validation, cells were resuspended in complete RPMI-1640 medium supplemented with 10% FBS and cultured at 37 °C for 48 h. Post-delivery membrane integrity was evaluated by propidium iodide (PI) staining (Solarbio, Beijing, China, C0080). After delivery and waiting for 30 min, cells in the Control and Experiment groups were stained with PI and were quantified by flow cytometry.

### 2.8. Contact Angle and Surface Free Energy (SFE)

Contact angles were obtained using the fixed drop method with an OCA 25 contact angle analyzer (DataPhysics Instruments GmbH, Filderstadt, Germany), which could measure contact angles from the drop image with a resolution of ±0.01°. Each contact angle measurement was repeated three times by depositing droplets (2 μL) to ensure accuracy and reproducibility.

SFE was calculated by the Owens–Wendt–Rabel–Kaelble (OWRK) method. Contact angles of distilled water and diiodomethane were measured on the samples and used to calculate SFE by solving the OWRK equations. All replicate contact-angle measurements obtained with water and diiodomethane were included in the OWRK analysis. The fitting procedure yielded a single model-derived SFE value for each surface rather than replicate-level SFE values. The surface-tension components used in the calculation are listed in Table 1.

### 2.9. Microwell Fabrication and AFM Measurements

Microwell chips for single-cell confinement were fabricated using standard soft lithography and PDMS replica molding. Briefly, SU-8 2010 (Kayaku Advanced Materials) was spin-coated onto cleaned silicon wafers at 3000 rpm to form the microwell mold. The coated wafers were soft-baked at 65 °C for 5 min and 95 °C for 10 min, exposed using a laser direct writing system according to the designed microwell pattern, post-exposure baked under the same conditions, and developed in PGMEA. The mold was then hard-baked at 150 °C for 5 min. The microwell structure was designed as an array of cylindrical microwells [41]. PDMS prepolymer and curing agent were mixed at a weight ratio of 10:1, degassed under vacuum, poured onto the SU-8 mold, and cured at 80 °C. The cured PDMS microwell layer was peeled from the mold and used for subsequent single-cell loading. Cells were loaded into the microwell chip before AFM (MFP-3D, Asylum Research, Oxford Instruments, Santa Barbara, CA, USA) measurements. To minimize the effects of surface hydrophobicity, all nanoneedle arrays used for AFM measurements were pre-wetted in ethanol for 5 min, rinsed 10 times with PBS, and finally equilibrated in RPMI-1640 medium before cell loading. The nanoneedle substrate and the cell-loaded microwell chip were transferred onto the same glass slide and immersed in RPMI-1640 medium (Gibco, 61870036) supplemented with 10% FBS (Gibco, A5670701). Tipless AFM cantilevers (SD-qp-SCONT-TL, NANOSENSORS, Neuchatel, Switzerland) were rinsed three times with ultrapure water (≈18 MΩ·cm), dried on precision wipes (Kimtech Science, Kimberly-Clark Professional, Roswell, GA, USA), and treated in an ultraviolet–ozone cleaner for 5 min. The cantilevers were then incubated in 10 mg/mL concanavalin A (MCE, HY-P2149A) at 37 °C for 1 h, rinsed with PBS, and immediately used for cell attachment. Before measurements, the inverse optical lever sensitivity was calibrated on a glass substrate, and the cantilever spring constant was determined using the Sader method [42]. To attach a single cell, the tipless cantilever was positioned above an individual cell confined in a microwell, pressed against the cell with a loading force of 1 nN, and held for 1 min. After successful cell attachment, the cantilever carrying the single cell was lifted from the microwell and maintained in the medium for 10 min to stabilize cell adhesion. During cell penetration experiments, the cell-attached cantilever was driven toward the nanoneedle array surface with an approach distance of 8 μm, an approach speed of 2 μm/s, and a trigger force of 80 nN. For each cell, force-indentation curves were acquired at 16 randomly selected positions on the nanoneedle surface. After each 16-point measurement, the cantilever was replaced, and a new cell was attached for the next penetration experiment.

### 2.10. Data Processing

Raw AFM force curves were exported using Igor Pro 6.3.1 (WaveMetrics, Lake Oswego, OR, USA) and analyzed using custom MATLAB R2020a (MathWorks, Natick, MA, USA) scripts. The force signal was baseline-corrected using the initial non-contact region of each approach curve. The baseline mean, μ, and standard deviation, σ, were calculated from this region, and the contact point was defined as the first position at which the corrected force exceeded μ+3σ and showed a sustained increase. Penetration events were identified from discontinuous force-drop features in the baseline-corrected force-indentation curves. Candidate events were detected when an abrupt negative force discontinuity exceeded the local baseline noise and was temporally localized within the approach curve. For each detected event, the event onset was defined as the local force maximum immediately before the discontinuity, and the event endpoint was defined as the subsequent local force minimum or relaxation point after the force drop. The force-drop magnitude was calculated as ΔF = |F_start_ − F_end_|. Events caused by baseline fluctuation, long-range drift, or unstable cell attachment were excluded during subsequent inspection. For each penetration-positive force curve, the first penetration event was defined as the earliest validated discontinuity along the indentation axis. The force and indentation at the onset of this first event were defined as the first penetration force, F_0_, and first penetration indentation, I_0_, respectively.

Penetration probability was calculated at the single-cell level. For each cell, 16 force curves were acquired at randomly selected positions on the nanoneedle surface, and the penetration probability was defined as the number of force curves containing at least one validated penetration event divided by the total number of tested force curves. For the spacing-force relationship, cell-level F_0_ values were calculated by averaging the F_0_ values of all penetration-positive force curves within each 16-curve cell map. The 1–5 μm nanoneedle arrays fabricated by one-step ICP etching were fitted using a phenomenological load-sharing model, F_0_ = F_∞_ + K/p^2^, where p is the nanoneedle spacing, F_∞_ is the fitted asymptotic force, and K is a density-dependent scaling coefficient. The fitting was performed by least-squares minimization of the residual sum of squares between the measured cell-level mean F_0_ values and the model-predicted values. Because the model is linear with respect to F_∞_ and K after defining x = 1/p^2^, the parameters were estimated by ordinary least squares. The model is used as a first-order phenomenological description and not as a complete contact-mechanics law. Data are presented as mean ± s.e.m.

## 3. Results

### 3.1. Rapid Fabrication of Spacing-Tunable Nanoneedle Arrays

**Figure 1:**
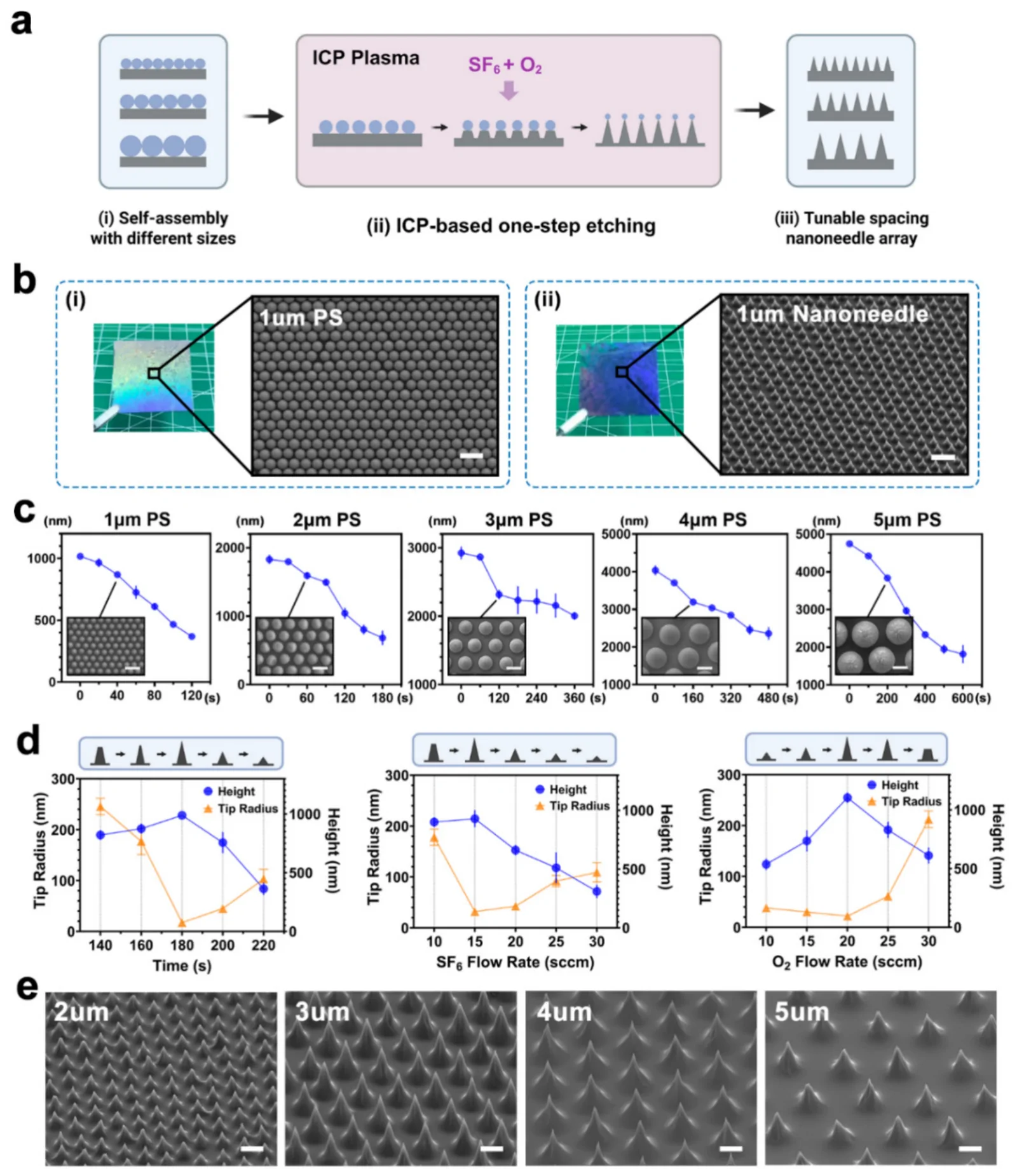
Rapid fabrication of spacing-tunable nanoneedle arrays via one-step etching. (**a**) Schematic of the fabrication process: (**i**) self-assembly using different PS microsphere sizes; (**ii**) ICP-based one-step etching; and (**iii**) formation of spacing-tunable nanoneedle arrays. (**b**) (**i**) Photograph and SEM image of a large-area 1 μm PS microsphere monolayer; (**ii**) photograph and SEM image of the resulting nanoneedle array. Scale bar, 2 μm. (**c**) Diameter evolution of 1–5 μm PS microspheres during O_2_ plasma etching. Insets, representative SEM images. Scale bar, 2 μm. (**d**) Effects of etching time, SF_6_ flow rate, and O_2_ flow rate on nanoneedle height and tip radius using 1 μm PS masks. (**e**) SEM images of nanoneedle arrays fabricated using 2–5 μm PS microspheres, showing tunable array spacing. Scale bar, 2 μm.

We developed a self-assembly-assisted fabrication strategy compatible with 4-inch-wafer processing for geometry-tunable silicon nanoneedle arrays by combining PS microsphere self-assembly with one-step etching (Figure 1a). In this process, PS microspheres with different diameters were first assembled into large-area close-packed monolayers under a gradient surface-tension-driven self-assembly process [40] and then transferred onto silicon substrates as sacrificial etching masks (Figure 1(ai)). After loading samples into the ICP chamber, the PS microspheres on the silicon substrate were first reduced in size by O_2_ plasma treatment to tune the exposed silicon area. A mixed SF_6_/O_2_ plasma was then introduced to etch the exposed silicon and generate nanoneedle arrays in a single step (Figure 1(aii)). Owing to the protection of the PS mask and simultaneous etching of the exposed silicon regions, the colloidal mask pattern was directly converted into ordered nanoneedle arrays, with the center-to-center spacing primarily determined by the initial microsphere diameter and the final morphology regulated by ICP parameters (Figure 1(aiii)). A large-area 1 μm PS self-assembly monolayer exhibited clear structural color under laboratory observation (Figure 1(bi)), indicating the formation of a highly ordered colloidal mask. After ICP etching, the chip surface exhibited an obvious color change and turned dark purple, consistent with the formation of micro/nanostructured silicon. SEM characterization confirmed that one-step etching produced ordered nanoneedle arrays (Figure 1(bii)).

To achieve precise geometry control over different nanoneedle spacing values, we first investigated the size evolution of PS microspheres under O_2_ plasma etching at a flow rate of 40 sccm (Figure 1c). For 1–5 μm PS microspheres, the diameter decreased with increasing O_2_ plasma exposure time (Figure S1). Reactive oxygen species generated in the plasma oxidize the PS surface, causing aromatic-ring opening and polymer-chain scission. Further oxidation produces volatile products, primarily CO and CO_2_, which are removed from the surface [43]. Ion bombardment assists bond cleavage and material removal from plasma-exposed regions, resulting in progressive shrinkage of the microspheres [44]. The O_2_ plasma etching time, therefore, controlled the residual PS mask size and the exposed silicon area for subsequent SF_6_/O_2_ etching. Insufficient shrinkage left oversized masks and produced plateau-like structures owing to limited silicon exposure, whereas excessive shrinkage over-consumed the masks, caused excessive silicon exposure, and ultimately led to over-etched, flattened nanostructures (Figure S2). By balancing silicon exposure and mask stability, the optimized O_2_ plasma etching times for 1, 2, 3, 4, and 5 μm PS microspheres were determined to be 40, 60, 120, 160, and 200 s, respectively (Figure 1c, inset). Using 1 μm PS monolayers as a representative, we systematically studied how ICP etching parameters control nanoneedle height and tip radius, including etching time, SF_6_ flow rate, and O_2_ flow rate (Figure 1d). When the SF_6_ and O_2_ flow rates were fixed, the increasing etching time progressively transformed the morphology from truncated-cone-like nanostructures to well-defined conical nanoneedles and finally to over-etched, shortened nanoneedles (Figure 1d). Correspondingly, the nanoneedle height initially increased and then decreased, whereas the tip radius showed an opposite trend. This morphology evolution reflects the competition among vertical silicon etching, lateral etching, and gradual consumption of the PS mask (Figure S3). At the early stage, anisotropic removal of the exposed silicon promoted nanoneedle elongation. With increased etching time, enhanced lateral etching and progressive PS mask degradation gradually sharpened the structures into conical nanoneedles. However, excessive etching caused severe sidewall erosion and mask loss, resulting in over-etched nanoneedles with reduced height. At a fixed etching time of 180 s, we further examined the effects of SF_6_ and O_2_ flow rates (Figure 1d). Within the tested SF_6_ flow range of 10–30 sccm, increasing the SF_6_ flow initially enhanced silicon removal and produced taller and sharper nanoneedles, but excessive SF_6_ led to over-etching, reduced anisotropy, and tip blunting. By contrast, insufficient O_2_ could not provide adequate sidewall protection, whereas excessive O_2_ diluted the SF_6_-driven etching component and promoted under-etched or more cylindrical morphologies (Figures S4 and S5). This trend is consistent with SF_6_/O_2_ plasma etching chemistry, in which fluorine radicals drive silicon volatilization, while oxygen regulates fluorine availability and promotes oxide-mediated surface passivation [45]. Therefore, the final nanoneedle profile is determined by the coupled evolution of the PS mask, vertical etching, and lateral sidewall erosion. After parameter optimization, ordered nanoneedle arrays with tunable spacing from 1 to 5 μm were fabricated under the optimized conditions (Figure 1b,e), establishing a versatile nanoneedle interface for subsequent mechanical characterization and intracellular delivery studies. Tapping-mode AFM images and high-magnification 45° tilted SEM images were also provided (Figures S6 and S7). EDS elemental mapping showed that the nanoneedle array was predominantly composed of Si (Figure S8). The map sum spectrum contained 99.75 at.% Si and 0.25 at.% O, with no other elemental signals detected within the analyzed area.

### 3.2. Fabrication of HAR Nanoneedle Arrays via Pseudo-Bosch Etching

**Figure 2:**
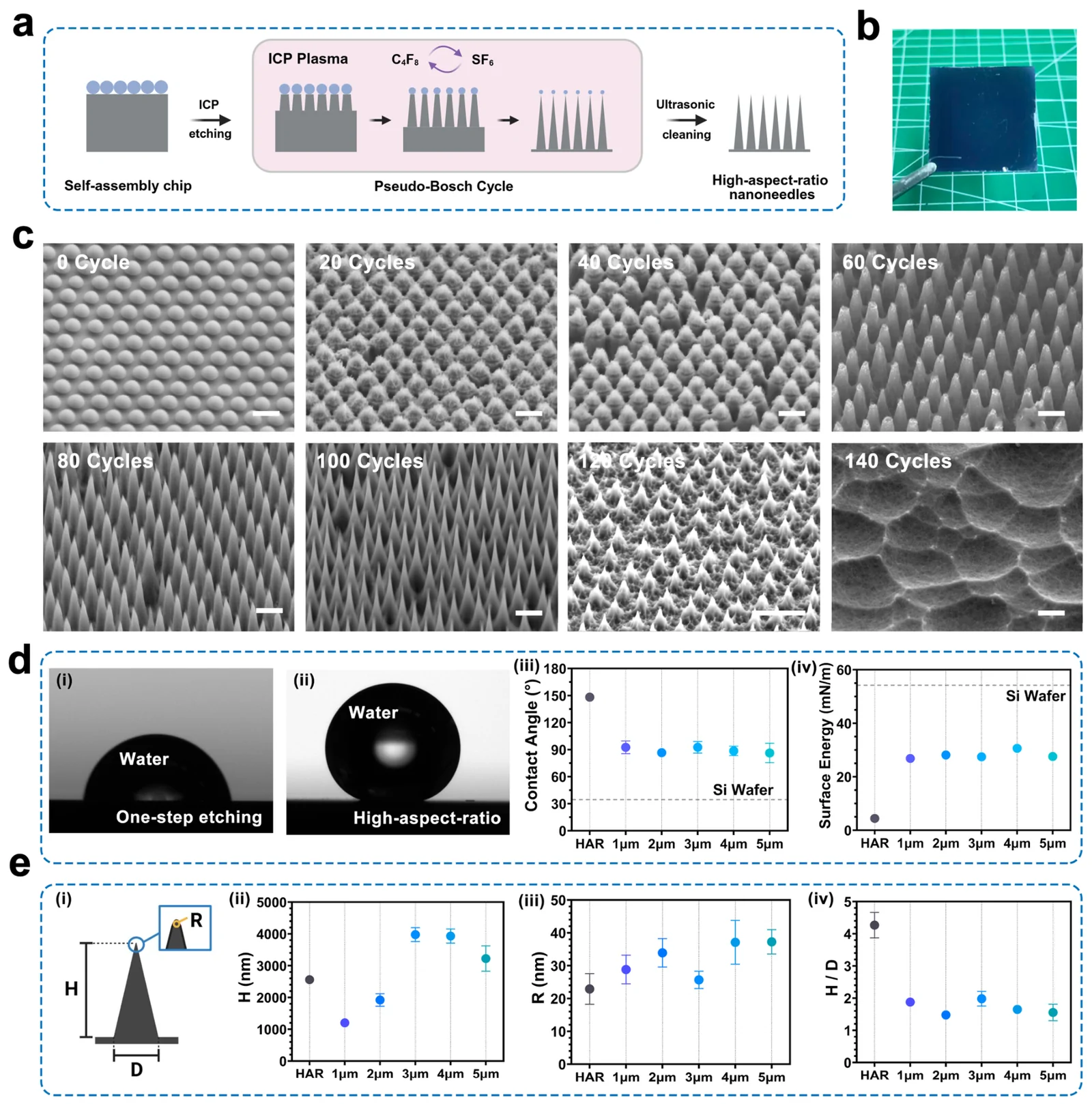
Fabrication of HAR nanoneedle arrays by pseudo-Bosch etching. (**a**) Schematic of pseudo-Bosch etching, including C_4_F_8_/SF_6_ etching cycles and ultrasonic cleaning. (**b**) Photograph of a HAR nanoneedle chip under laboratory conditions. (**c**) SEM images of PS-masked silicon surfaces from 0 to 140 C_4_F_8_/SF_6_ etching cycles. Scale bar, 1 μm. (**d**) Surface wettability characterization: (**i**) water contact angle of a one-step-etching array; (**ii**) water contact angle of the HAR array; (**iii**) water contact angles across the silicon wafer, HAR array, and one-step-etching arrays; and (**iv**) calculated SFE values across the same surfaces. SFE was determined by fitting the OWRK model to all replicate contact-angle measurements for each surface. Aspect ratios are presented as mean ± SD of individual-nanoneedle H/D values. (**e**) Quantitative geometry analysis: (**i**) definitions of nanoneedle height (H), basal diameter (D), and tip radius (R); (**ii**) nanoneedle height; (**iii**) tip radius; and (**iv**) aspect ratio (H/D) for the HAR array and one-step-etching arrays with different spacing values.

To further increase the nanoneedle aspect ratio, we developed pseudo-Bosch etching to fabricate HAR nanoneedle arrays (Figure 2a). In this strategy, the self-assembled PS microsphere monolayer was similarly used as a sacrificial mask, while alternating C_4_F_8_ passivation and SF_6_ etching steps were introduced to enhance etching anisotropy. In this cyclic process, the C_4_F_8_ step provides fluorocarbon-based sidewall protection to suppress lateral erosion, while the SF_6_ step supplies reactive fluorine species for vertical silicon etching. As the PS mask was progressively consumed during cyclic etching, the final HAR morphology resulted from a dynamic balance among mask shrinkage, sidewall passivation, and vertical silicon etching, ultimately producing a dark silicon surface under the optimized experimental conditions (Figure 2b). SEM characterization showed a clear cycle-dependent morphology evolution (Figure 2c). Before cyclic etching, PS microspheres formed a close-packed monolayer on the silicon surface. After 20–40 cycles, the structures evolved into relatively broad and short column-like structures. At approximately 60 cycles, the top region began to shrink, and the PS mask was largely consumed, leading to truncated cone-like structures. Needle-like morphologies became evident after 80 cycles, and well-defined HAR nanoneedles with improved height and sharpness were obtained after 100 cycles. Increasing the cycle number to 120 led to roughened and partially degraded structures, whereas 140 cycles resulted in excessive etching. We further summarized the nanoneedle height and tip radius over different pseudo-Bosch etching cycles (Figure S9). As the cycle number increased from 20 to 100, the estimated height increased from 0.699 to 2.727 μm, whereas the tip radius decreased from 587.7 to 22.9 nm. At 120 cycles, the height decreased markedly to 1.107 μm and the tip radius increased slightly, indicating structural degradation due to over-etching. At 140 cycles, the nanoneedle structures were completely removed. Therefore, 100 cycles was selected as the optimized parameter for HAR nanoneedles. SEM images obtained from randomly selected locations across the wafer showed similar nanoneedle morphologies (Figure S10). Such consistency was achieved via a uniform PS mask and plasma distribution within the ICP chamber. This cycle-dependent evolution confirms that pseudo-Bosch etching can enhance anisotropy within an optimized processing window.

We next compared the surface wetting properties of all nanoneedle arrays (Figure 2d). Nanoneedle arrays fabricated by one-step etching with different spacing values showed water contact angles of approximately 90°. In contrast, the HAR nanoneedle arrays exhibited a water contact angle of approximately 150°, indicating superhydrophobic behavior (Figure 2(di,dii)). An untreated silicon wafer showed a much lower contact angle of approximately 31° and served as the control group (Figure 2(diii)). Consistently, the calculated SFE of the HAR nanoneedle surface was lower than that of arrays fabricated by one-step etching and the silicon wafer (Figure 2(div)). We further quantified and compared the key parameters of the different nanoneedle arrays (Figure 2e). Compared with nanoneedles fabricated by one-step etching using the same 1 μm PS microspheres, the HAR nanoneedles exhibited substantially increased height, reduced tip radius, and a larger aspect ratio. The aspect ratio of the HAR nanoneedles reached approximately 4, nearly twice that of the arrays with 1–5 μm spacing fabricated by one-step etching. Together, these results demonstrate that pseudo-Bosch etching expands the accessible nanoneedle morphology by balancing vertical etching with sidewall protection, enabling large-area HAR arrays with increased aspect ratio, sharper tips, and distinct wetting properties. To facilitate a direct comparison of the two fabrication methods, the characteristics of one-step etching and pseudo-Bosch etching are further summarized in Table 2.

### 3.3. Nanoneedle Spacing Regulates Single-Cell Penetration Mechanics

**Figure 3:**
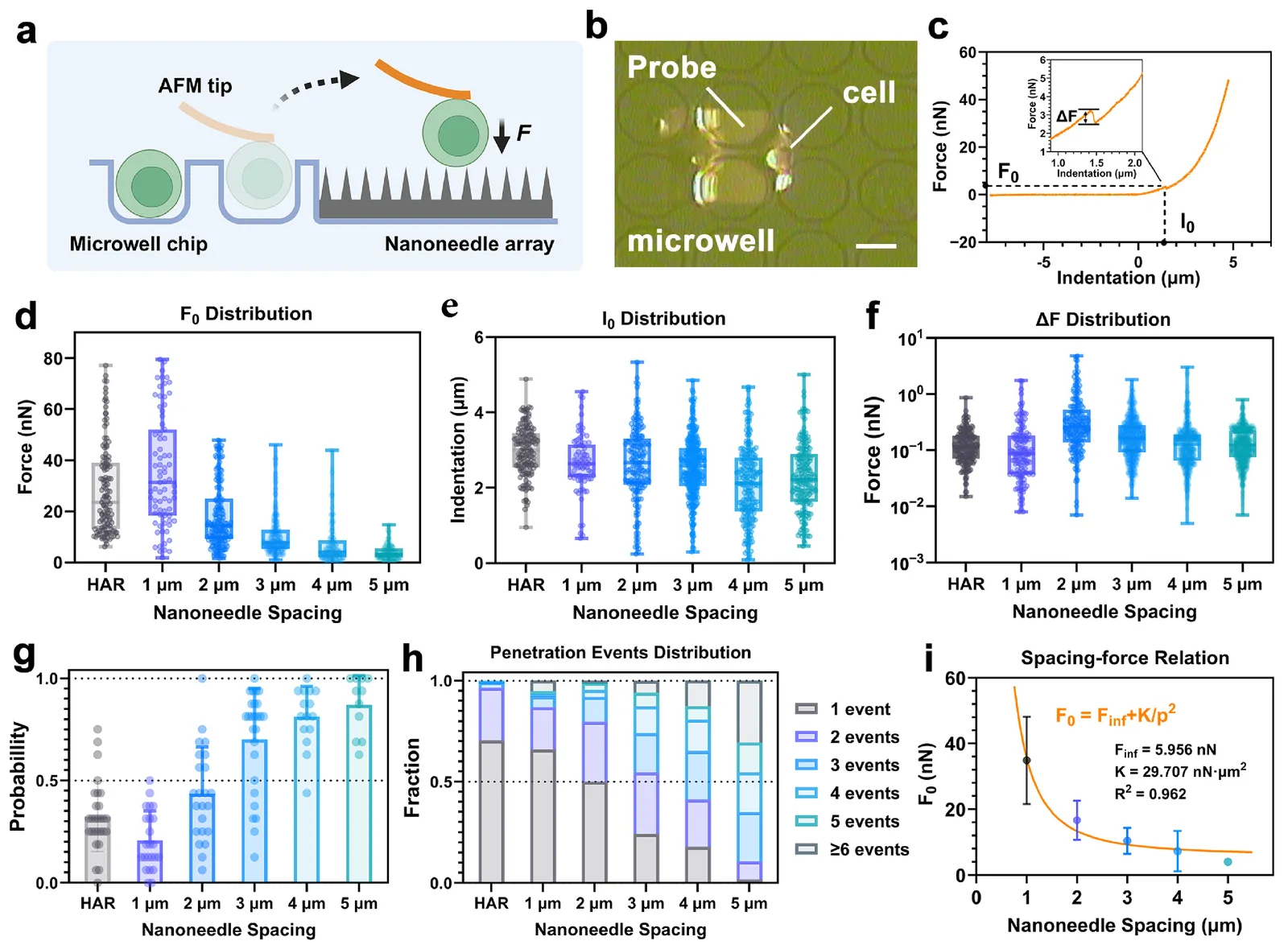
Nanoneedle spacing regulates membrane penetration mechanics. (**a**) Schematic of the AFM-based membrane penetration analysis workflow, in which single cells confined in microwells were brought into contact with nanoneedle arrays by a tipless probe. The dashed arrow indicates movement of the cell-attached AFM probe toward the nanoneedle array. (**b**) Representative optical image showing cell attachment to the AFM probe. Scale bar, 20 μm. (**c**) Representative force–indentation curve with a membrane penetration event (saw-tooth pattern). First penetration force, F_0_. First penetration indentation, I_0_. Force-drop magnitude, ΔF. (**d**,**e**) Distributions of F_0_ and I_0_ for HAR nanoneedles and nanoneedle arrays with 1–5 μm spacing fabricated by one-step etching. (**f**) Distribution of ΔF values plotted on a logarithmic scale. (**g**) Penetration probability calculated from repeated single-cell force measurements, with each cell measured at 16 random positions. (**h**) Distribution of penetration-event numbers among penetration-positive force curves for different nanoneedle arrays. (**i**) Force–spacing relationship for arrays with 1–5 μm spacing fabricated by one-step etching fitted with F_0_ = F_∞_ + K/p^2^, where p is nanoneedle spacing. HAR, *N* = 27 cells; 1 μm, *N* = 21 cells; 2 μm, *N* = 23 cells; 3 μm, *N* = 26 cells; 4 μm, *N* = 15 cells; 5 μm, *N* = 11 cells.

To quantify how nanoneedle-array geometry influences the membrane penetration process, we established a cell-on-probe AFM platform for single-cell studies (Figure 3a). Individual cells were first confined in microwells and then gently contacted by a tipless AFM probe functionalized with adhesive protein. A small preload of approximately 1 nN was applied to attach a single cell to the probe surface (Figure 3b). The cell-attached probe was then lowered to indent the attached cell on nanoneedle surfaces. Membrane penetration events appeared as saw-tooth patterns with discrete force drops (Figure 3c). We defined the first penetration force (F_0_), the corresponding indentation depth (I_0_), and the penetration-associated force-drop magnitude (ΔF) from the force–indentation curve. Using this workflow, we systematically analyzed the penetration mechanics of the nanoneedle arrays fabricated above (Figure 3d–i). To minimize wetting-related artifacts, the nanoneedle substrates were first fully infiltrated with ethanol, followed by repeated rinsing with PBS. The buffer was then replaced with complete culture medium for subsequent cell attachment and mechanical studies. Bright-field images of the same cantilever-bound Jurkat E6-1 cell showed no visible fragmentation after the cell penetration test (Figure S11). Descriptive statistics for the cell-level F_0_ values are provided in Table S1. Across the arrays with 1–5 μm spacing fabricated by one-step etching, cell-level F_0_ showed a strong negative monotonic association with nanoneedle spacing (Spearman *ρ* = −0.825, *p* = 4.94 × 10^−25^; Table S2). For nanoneedle arrays fabricated by one-step etching, F_0_ decreased progressively from 34.87 ± 2.90 nN at 1 μm spacing to 16.68 ± 1.24, 10.41 ± 0.78, 7.25 ± 1.59, and 4.05 ± 0.30 nN at 2, 3, 4, and 5 μm spacing, respectively (Figure 3d). The trend was monotonic across the tested spacing values. In comparison, the HAR nanoneedles with 1 μm spacing showed an intermediate F_0_ distribution of 25.90 ± 2.50 nN compared with the arrays with 1 and 2 μm spacing fabricated by one-step etching, suggesting that increasing the aspect ratio and reducing the tip radius facilitate membrane penetration but do not fully eliminate the density-dependent load-sharing effect of nanoneedle arrays. In contrast to F_0_, the first penetration indentation I_0_ and the force-drop magnitude ΔF varied within narrower ranges across different arrays. The mean I_0_ values remained around 2–3 μm under most conditions, while most ΔF values fell within the 10^−1^ to 10^0^ nN range (Figure 3e,f).

Under identical force-curve acquisition settings, membrane penetration was not deterministic but occurred with a probability that depended on nanoneedle-array geometry. We therefore quantified cell-level penetration probability and penetration-event number distributions for different nanoneedle arrays (Figure 3g,h). For nanoneedle arrays fabricated by one-step etching, penetration probability increased with spacing, rising from 0.21 ± 0.03 at 1 μm to 0.43 ± 0.05, 0.70 ± 0.05, 0.81 ± 0.04, and 0.87 ± 0.04 at 2, 3, 4, and 5 μm, respectively. HAR arrays exhibited a penetration probability of 0.32 ± 0.03, higher than arrays with 1 μm spacing fabricated by one-step etching but lower than arrays with larger spacing (Table S2). Consistently, the number of penetration events for force–indentation measurements increased with spacing (Figure 3h). The mean event number increased from 1.88 events per positive curve at 1 μm to 4.58 events per positive curve at 5 μm, indicating that sparse arrays promote multiple sequential penetration events during a single indentation process. To describe the force–spacing trend, the group-mean F_0_ values for arrays with 1–5 μm spacing fabricated by one-step etching were fitted using the density-based phenomenological model F_0_ = F_∞_ + K/p^2^ (Figure 3i). The p^−2^ dependence was prescribed by the nominal density of an ideal hexagonal array rather than selected as the best empirical power law. The fit yielded F_∞_ = 5.956 nN, K = 29.707 nN·μm^2^, and R^2^ = 0.962. This relationship provides a first-order description of density-associated load sharing but does not establish spacing as the sole determinant because nanoneedle height, tip radius, aspect ratio, and wettability also varied across the arrays. The geometric basis and assumptions of the density-scaling model are detailed in Supplementary Note S1.

### 3.4. Nanoneedle Array Geometry Influences Vibration-Assisted Intracellular Delivery

**Figure 4:**
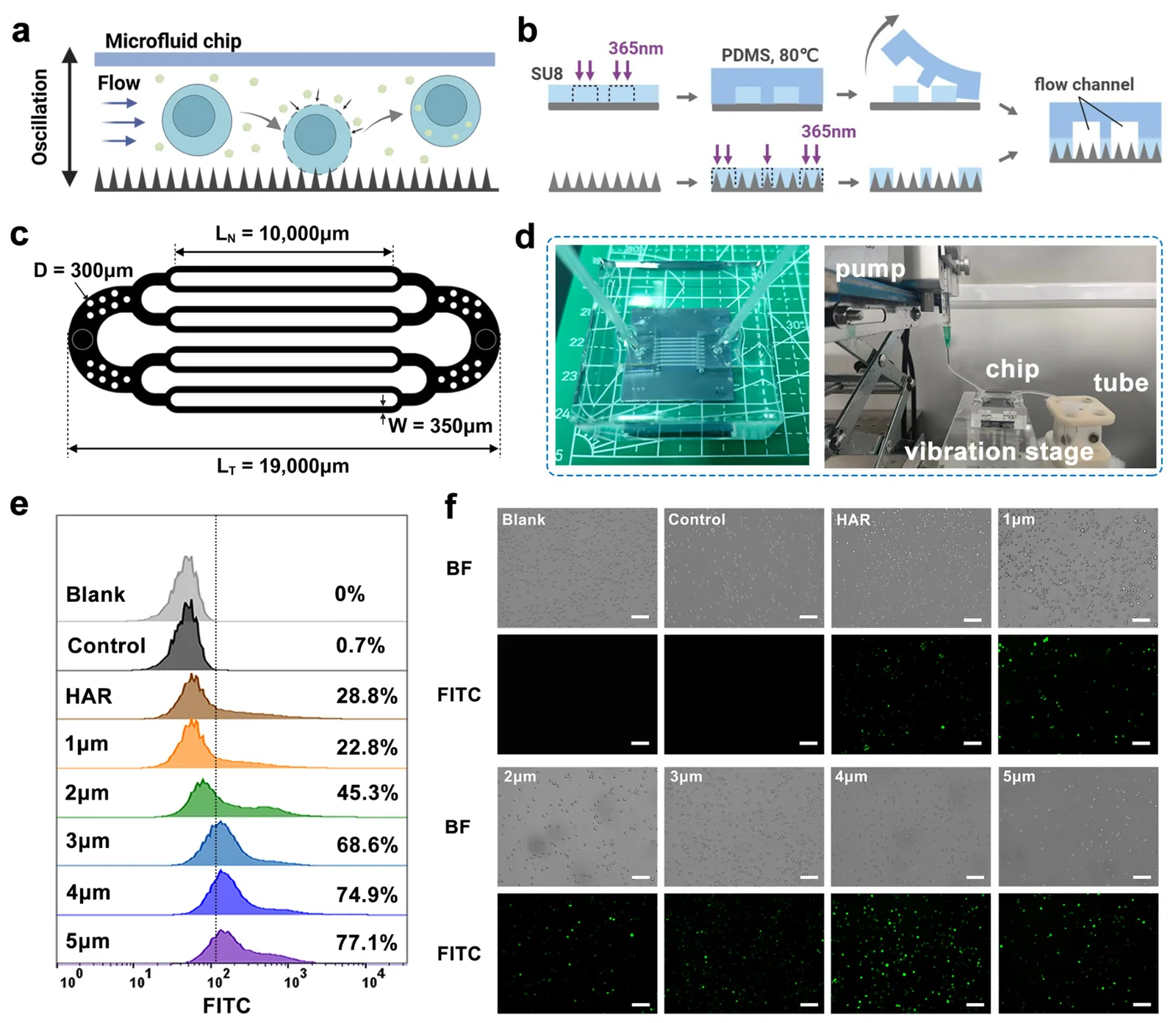
Nanoneedle array geometry affects vibration-assisted intracellular delivery. (**a**) Schematic of continuous-flow delivery through an oscillating nanoneedle-microfluid chip. (**b**) Fabrication methodology of the nanoneedle-microfluid chip. (**c**) Microchannel design and dimensions. (**d**) Photographs of the fabricated nanoneedle-microfluid chip and the microfluid platform assembly. (**e**) Flow cytometry histograms of FITC fluorescence in Jurkat E6-1 cells treated with HAR nanoneedles or arrays with 1–5 μm spacing fabricated by one-step etching. Dashed line, FITC-positive threshold. Percentage, FITC-positive cell fractions. Blank and Control groups served as negative references. (**f**) Bright-field and FITC fluorescence images of cells corresponding to the groups shown in the flow cytometry panel. Scale bar, 100 μm.

We integrated the nanoneedle chip with a microfluidic channel and applied vibration to compare delivery across array geometries (Figure 4a). The microfluidic device was fabricated using SU-8 photolithography and PDMS soft lithography (Figure 4b). In this process, a PDMS microchannel was replicated from an SU-8 master, while an additional SU-8 layer was patterned on the nanoneedle substrate with a bonding region and exposed nanoneedle area. The PDMS channel was then aligned with and plasma-bonded to the substrate, forming a sealed nanoneedle-microfluidic chip. The microchannel had a total length *L_T_* = 19,000 μm and a height *H* = 17 μm. The central region of the microchannel comprised eight parallel segments positioned above the exposed nanoneedle array, each with a width *W* = 350 μm and an effective nanoneedle-interaction length *L_N_* = 10,000 μm (Figure 4c). Finally, the chip was encapsulated in epoxy resin, connected to a syringe pump and collection tube, and mounted on a vibration stage to establish a continuous-flow intracellular delivery platform (Figure 4d).

For the delivery experiments, Jurkat E6-1 cells with a mean diameter of approximately 12 μm were suspended at 2 × 10^6^ cells/mL in PBS containing 10 kDa FITC–dextran and introduced into the microchannel at a flow rate of 200 μL/min. All experiments were performed under identical sinusoidal vibration conditions, with an acceleration *a* = 8 g and a frequency *f* = 50 Hz. The vertical oscillation induced repeated cell–nanoneedle contact, facilitating membrane permeabilization and cargo entry. The collected cells were then incubated for 20 min, washed with PBS, and analyzed by flow cytometry. The flow cytometry results showed a clear dependence of FITC-dextran delivery on nanoneedle geometry (Figure 4e). The Blank and Control groups showed FITC-positive fractions of 0 and 0.7%, whereas all nanoneedle-treated groups exhibited right-shifted fluorescence distributions. For the nanoneedle arrays fabricated by one-step etching, the FITC-positive fraction increased with spacing from 22.8% at 1 μm to 45.3%, 68.6%, 74.9%, and 77.1% at 2, 3, 4, and 5 μm, respectively. The increase was most pronounced between 1 and 3 μm, whereas the difference between the 4 and 5 μm arrays was relatively small, indicating that delivery approached a plateau under the tested conditions. This trend was consistent with the spacing-dependent decrease in first penetration force F_0_ observed in the AFM measurements (Figure 3i). At the same nanoneedle spacing of 1 μm, the HAR arrays achieved a higher FITC-positive fraction than the arrays fabricated by one-step etching, at 28.8% versus 22.8%. Nevertheless, the delivery efficiency of the HAR arrays remained lower than that of the arrays with 2–5 μm spacing fabricated by one-step etching. Bright-field and FITC fluorescence images showed the same overall trend, with minimal fluorescence in the Blank and Control groups and progressively more fluorescent cells in the groups with larger nanoneedle spacing (Figure 4f). Similarly, we confirmed that cells treated using the nanoneedle-microfluidic chip could be further cultured while maintaining intracellular fluorescence (Figure S12). The cell viability of the experimental group did not differ markedly from that of the control group via PI assay testing (Figure S13). Overall, intracellular delivery increased with nanoneedle spacing and was modestly enhanced by the HAR morphology at the matched 1 μm spacing. Under the current operating conditions, array spacing therefore exerted a stronger influence on delivery efficiency than nanoneedle morphology alone.

## 4. Discussion

This work establishes a self-assembly-assisted ICP etching strategy for the rapid fabrication of large-area silicon nanoneedle arrays with tunable geometry, combined with membrane penetration mechanics and intracellular delivery validation. Compared with conventional photolithography-based fabrication, self-assembled PS microspheres serve as both spacing-defining templates and sacrificial masks, eliminating the need for expensive exposure systems and complex lithography. The PS mask is progressively consumed during etching, enabling rapid nanoneedle formation by one-step etching without additional tip sharpening. Final morphology is governed by the coupled evolution of the PS mask and silicon substrate during SF_6_/O_2_ ICP etching. SF_6_ promotes silicon removal through volatile SiF_x_ formation, whereas O_2_ regulates vertical etching and lateral erosion through oxygen-derived surface passivation [46,47]. Oxygen adsorption and fluorinated oxide formation can suppress lateral etching and improve anisotropy, although excessive oxygen can reduce the vertical etch rate by passivating active bottom sites [48]. This mechanism is consistent with our observations: insufficient PS shrinkage limits silicon exposure, whereas excessive mask consumption or unbalanced SF_6_/O_2_ ratios promote lateral erosion, over-etching, or flattened structures. Within an optimized process window, the balance among mask shrinkage, vertical etching, and sidewall protection yields well-defined conical nanoneedles.

In one-step etching, the nanoneedle aspect ratio is limited by the coupled effects of SF_6_/O_2_ plasma etching and PS-mask consumption. Increasing the aspect ratio therefore requires independent control of sidewall protection and silicon removal. We used pseudo-Bosch etching as a complementary route for HAR nanoneedle fabrication. C_4_F_8_ provides fluorocarbon-based sidewall passivation, whereas SF_6_ supplies reactive fluorine species for vertical etching. HAR morphology depended on cycle number and was obtained within a limited process window: insufficient cycles produced broad structures, whereas excessive cycles consumed the mask and degraded the nanoneedles. The HAR arrays also showed black-silicon-like optical absorption and superhydrophobicity, which may support applications in light trapping, photothermal conversion, self-cleaning, and antifouling [49,50,51]. For intracellular delivery, however, this wetting behavior may affect liquid infiltration, protein adsorption, and cell–nanoneedle contact under microfluidic conditions. Compared with lithography-based methods, our strategy prioritizes process simplicity and scalable fabrication. Stepper or electron-beam lithography combined with deep reactive ion etching and post-etch sharpening can provide more deterministic patterning and higher aspect ratios [27,28], but requires additional processing. By extending our previous fixed-geometry self-assembled-mask method [36], one-step etching generated 1–5 μm spacing arrays across 4-inch wafers, while pseudo-Bosch etching produced a 1 μm spacing HAR array. The approach is suited to comparative geometry studies, although spacing selection and uniformity remain constrained by microsphere self-assembly and the plasma process window.

The AFM results indicate that nanoneedle-array penetration involves load sharing. Unlike single-nanoneedle AFM [32,33,34] or array studies based on imaging, modeling, or delivery outcomes [29,30,31,35,36,37,38], the cell-on-probe experiments analyzed array-level force curves and repeated penetration events during simultaneous multi-tip contact. The higher first penetration force observed for denser arrays is consistent with a “bed-of-nails” effect, in which the applied load is distributed across more tips [52,53]. For an ideal hexagonal array, the number of nanoneedles within a fixed area scales with 1/p^2^; under the simplified assumptions of an approximately constant effective contact area, engaged-tip fraction, and mean local penetration threshold, this gives F_0_ = F_∞_ + K/p^2^. The inverse-square relationship is therefore a first-order phenomenological description rather than a first-principles contact-mechanics model. The Hertz model can describe elastic deformation before penetration, but its assumptions become restrictive for sharp probes and nonlinear effects [54]. The JKR model incorporates adhesion at a single contact, yet application to a multi-contact interface requires additional assumptions regarding the distribution of asperities or contacts [55], and neither model directly describes membrane rupture. Recent finite-element models of nanoneedle penetration account for membrane-cortex mechanics, cytoplasmic response, tip geometry, and a rupture criterion [56], while continuum membrane models show that membrane bending and tension affect the resistive force generated by needle-like structures [57]. Overall, developing a predictive model of the interaction between a single cell and multiple nanoneedles remains an important challenge. At the same 1 μm spacing, the lower penetration force and higher penetration probability of the HAR array indicate an additional contribution from nanoneedle morphology, suggesting that arrays with higher aspect ratios and sharper tips facilitate cell membrane penetration. However, HAR arrays did not fully reproduce the low penetration forces of larger-spacing arrays, suggesting that array spacing remains a dominant parameter for membrane penetration.

The intracellular delivery experiments further supported the geometry-associated differences in membrane penetration. A channel height of 17 μm was selected for Jurkat E6-1 cells (mean diameter, approximately 12 μm) to avoid excessive confinement and preserve sensitivity to differences among nanoneedle arrays [35]. Under fixed flow and vibration conditions, the FITC–dextran-positive fraction increased from 22.8% for the 1 μm array to 45.3%, 68.6%, 74.9%, and 77.1% for the 2, 3, 4, and 5 μm arrays fabricated by one-step etching, respectively. The HAR array achieved 28.8% at the same 1 μm spacing, consistent with its lower first penetration force and higher penetration probability. Because height, tip radius, aspect ratio, and wettability co-varied across arrays, these results establish a geometry-associated trend rather than a spacing-specific effect. In the present study, 10 kDa FITC–dextran was used as a membrane-impermeable model macromolecular cargo rather than a pharmacologically active drug. Oscillating nanoneedles achieved 34% plasmid expression and 42% Cre recombination in adherent cells [27], whereas a herringbone/nanoneedle flow device reported >20% gene transfection with >95% viability [38]. Our earlier vibration-assisted system delivered Cas9 ribonucleoproteins to immune cells with efficiencies up to 98% and approximately 80% viability [36], and a piezoelectric–nanoneedle platform achieved 83.1–94.6% FITC–dextran-positive fractions under cell-specific optimized conditions [35]. Other microfluidic mechanoporation approaches reported 44–65% delivery of 70 kDa dextran [58] and delivery efficiencies up to approximately 95% under optimized conditions [59]. Here, the FITC–dextran-positive fraction ranged from 22.8% to 77.1% under one fixed operating condition. These comparisons provide a contextual benchmark rather than a direct performance ranking because the reported cell types, cargoes, actuation modes, and endpoints differ. Nevertheless, the force–spacing relationship and delivery outcomes reported here were established only in Jurkat E6-1 cells and should not be directly extrapolated to other cell types without further validation. Although our previous work demonstrated that continuous-flow vibration-assisted nanoneedle delivery could be adapted to different adherent and suspension cell types [35], and similar universality has been reported for constriction- and fluid-shear-based mechanoporation [58,59], differences in membrane composition and cellular mechanics still alter cell deformation and penetration mechanics. Extending the present findings to other cell types will therefore require further experimental validation and careful mechanistic analysis.

## 5. Conclusions

We developed a rapid, large-scale method for fabricating silicon nanoneedle arrays with tunable spacing and morphology, including arrays with 1–5 μm spacing fabricated by one-step etching and HAR arrays fabricated by pseudo-Bosch etching. Using a microwell-assisted cell-on-probe AFM platform, we quantified single-cell penetration mechanics and found that, within the one-step etching series, increased spacing was associated with lower first penetration forces, higher penetration probability, and higher delivery efficiencies in Jurkat E6-1 cells. The force–spacing trend was described by a phenomenological inverse-square model. These findings link nanoneedle geometry to single-cell penetration mechanics and delivery outcomes, providing mechanistic understanding for nanoneedle-based delivery and biosensor design. The rapid fabrication method may also facilitate broader applications such as light trapping in photovoltaic devices, photothermal conversion, and self-cleaning. Future work should combine refined mechanical modeling with higher-resolution, real-time single-cell analysis to determine how nanoneedle geometry and interfacial properties govern membrane deformation and penetration.

## Figures and Tables

**Table 1:** Test liquids and their surface tensions.

Surface Tension Data (mJ/m^2^)	*γ*_l_	*γ*_l_^d^	*γ*_l_^p^
Water, H_2_O	72.1	19.9	52.2
Diiodomethane, CH_2_I_2_	50.0	47.4	2.6

**Table 2:** Comparison of the one-step etching and pseudo-Bosch etching methods.

Category	Feature	One-Step Etching (SF_6_/O_2_)	Pseudo-Bosch Etching (C_4_F_8_/SF_6_)
**Process characteristics**	Etching sequence	Continuous SF_6_/O_2_ etching after O_2_-mask shrinkage	Alternating C_4_F_8_ passivation (10 s) and SF_6_ etching (8 s); 100 cycles
	Primary process controls	Mask-shrink time, SF_6_/O_2_ ratio, RF power, and etching time	Cycle number, step duration, gas flow, and RF/ICP power
**Structural characteristics**	Morphology	Shorter conical nanoneedles	Taller and sharper high-aspect-ratio nanoneedles
	Aspect ratio	Approximately 2	Approximately 4
**Interfacial property**	Water contact angle	Approximately 90°	Approximately 150° (superhydrophobic)
**Strengths and limitations**	Strength	Low process time, simple process, only RF power required	Greater height, sharpness, and aspect ratio
	Limitation	Height and sharpness are coupled to PS-mask consumption and lateral erosion	Process complexity and structural degradation after excessive cycles
**Applicable scenarios**	Applications in this study	Cell–nanoneedle penetration mechanics and continuous-flow intracellular delivery	Cell–nanoneedle penetration mechanics and delivery experiments
	Potential applications *	Rapid, low-cost, large-area fabrication approach for biointerfaces	Superhydrophobic surfaces and black-silicon-like optical, photothermal, or self-cleaning surfaces

* Potential suitability is inferred from nanoneedle geometry and prior literature; these applications were not directly tested in this study.

## Data Availability

The data presented in this study are available in the article and the Supplementary Materials. Additional data are available from the corresponding authors upon reasonable request.

## Supplementary Materials

The following supporting information can be downloaded at: https://www.mdpi.com/article/10.3390/bios16090495/s1, Figure S1: Time-dependent O_2_ plasma shrinkage of 1 μm PS microspheres; Figure S2: Effect of residual PS mask size on one-step-etched nanoneedle formation; Figure S3: Effect of etching time on nanoneedle morphology during one-step SF_6_/O_2_ ICP etching; Figure S4: Effect of SF_6_ flow rate on nanoneedle morphology during one-step ICP etching; Figure S5: Effect of O_2_ flow rate on nanoneedle morphology during one-step ICP etching; Figure S6: Tapping-mode AFM characterization of the one-step-etched nanoneedle array with 1 μm spacing; Figure S7: High-magnification tilted-view SEM images of the fabricated silicon nanoneedle arrays; Figure S8: Elemental characterization of a representative one-step-etched silicon nanoneedle array with 1 μm spacing by energy-dispersive X-ray spectroscopy (EDS); Figure S9: Evolution of HAR nanoneedle morphology with pseudo-Bosch etching cycles; Figure S10: Spatial morphology of the high-aspect-ratio silicon nanoneedle array at three sampling locations; Figure S11: Representative bright-field images of the same cantilever-bound Jurkat E6-1 cell; Figure S12: Fluorescence observation of Jurkat E6-1 cells after 48 h of post-delivery culture; Figure S13: Flow-cytometry plots of propidium iodide (PI)-stained Jurkat E6-1 cells after delivery experiments; Table S1: Descriptive statistics of cell-level first membrane-penetration forces across different nanoneedle arrays; Table S2: Statistical analysis of spacing- and morphology-dependent membrane penetration; Supplementary Note S1: Geometric basis of the nanoneedle array density-scaling model.
