# Supplementary material for: Geometry-Tunable Nanoneedle Arrays Reveal Membrane Penetration Mechanics for Intracellular Delivery
Source: Biosensors (Basel). 2026 Sep 4;16(9):495. doi: 10.3390/bios16090495 (PMC13604426; doi:10.3390/bios16090495)
Supplement: Supplementary file 1 [file biosensors-16-00495-s001.zip › biosensors-4508266-supplementary.pdf]

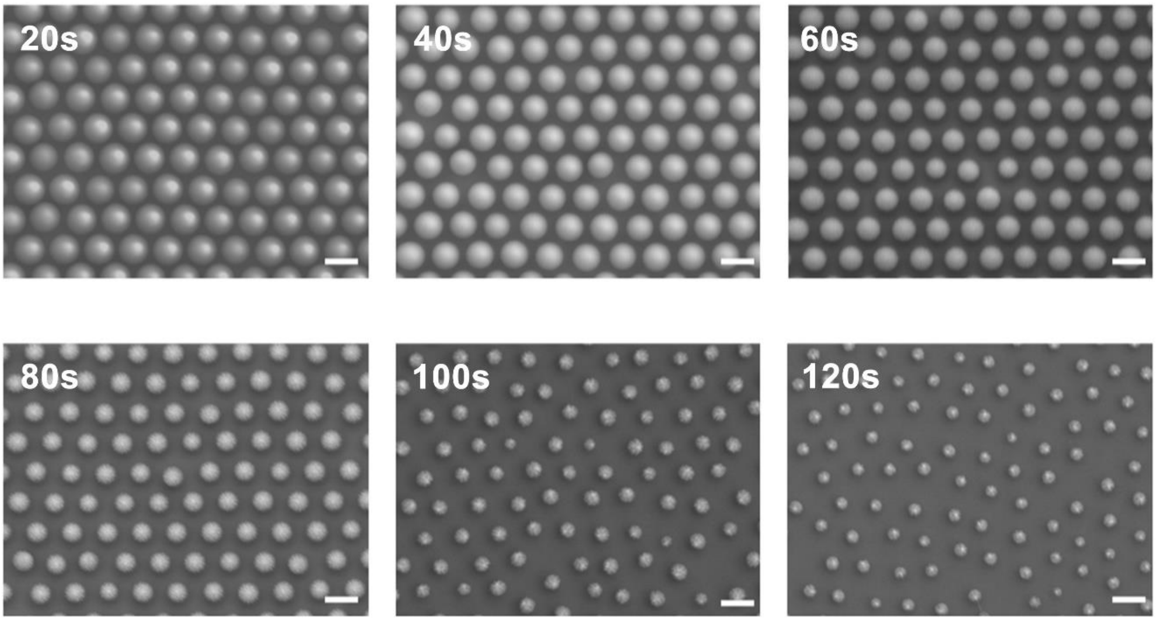

Figure S1. Time-dependent O_2 plasma shrinkage of 1 μm PS microspheres. Top-view SEM images of 1 μm PS microsphere monolayers after O_2 plasma treatment for 20, 40, 60, 80, 100, and 120 s at a fixed O_2 flow rate of 40 sccm. The progressive reduction in microsphere diameter increased the exposed silicon area available for subsequent SF_6/O_2 etching. Scale bars: 1 μm.

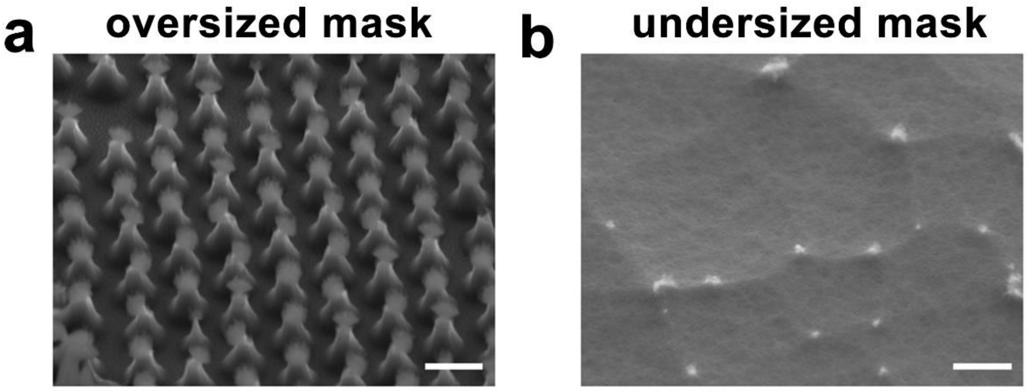

Figure S2. Effect of residual PS mask size on one-step-etched nanoneedle formation. SEM images of silicon substrates patterned with 1 μm PS microspheres after different O_2 plasma shrinkage times and subsequent SF_6/O_2 etching under otherwise identical conditions. **(a)** Oversized residual masks obtained after 20 s of O_2 plasma treatment limited silicon exposure and resulted in incompletely developed, broad nanostructures. **(b)** Undersized residual masks obtained after 120 s were rapidly consumed during silicon etching, causing excessive silicon exposure and severe over-etching of the surface. Scale bars: 1 μm.

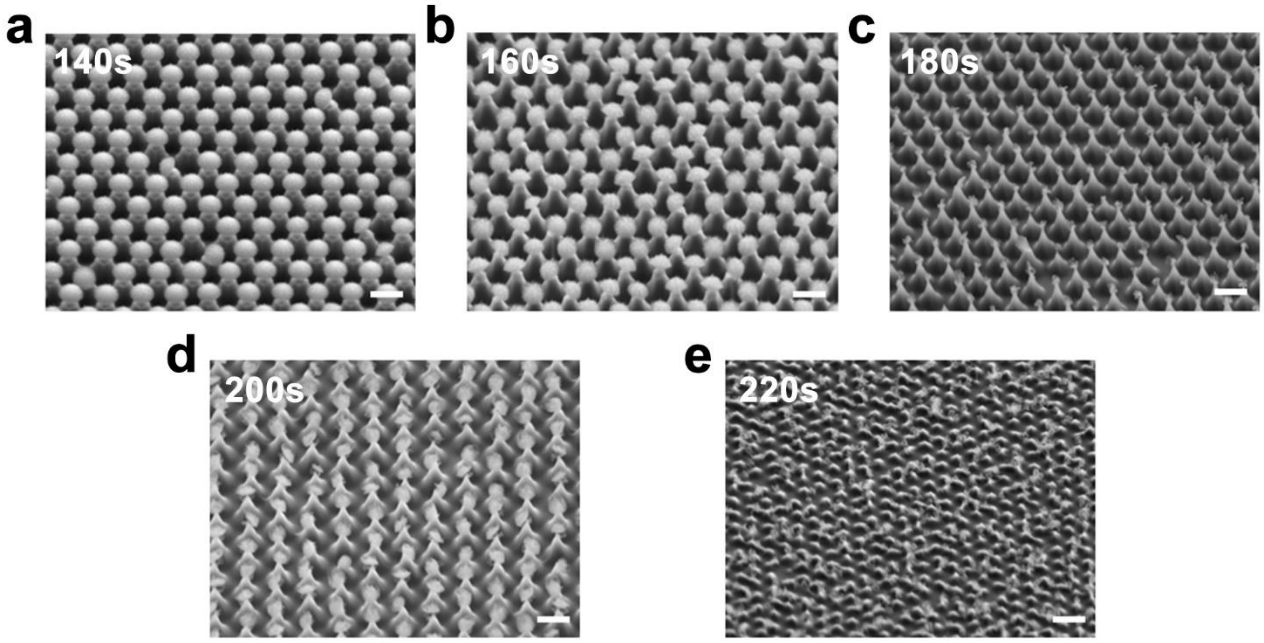

Figure S3. Effect of etching time on nanoneedle morphology during one-step SF_6/O_2 ICP etching. SEM images of nanostructures fabricated using 1 μm PS microsphere masks after etching for **(a)** 140, **(b)** 160, **(c)** 180, **(d)** 200, and **(e)** 220 s at fixed SF_6 and O_2 flow rates of 15 and 20 sccm, respectively. Increasing etching time progressively transformed the structures from broad, truncated-cone-like features into well-defined conical nanoneedles. Further etching caused mask loss, sidewall erosion, and over-etched morphologies. Scale bars: 1 μm.

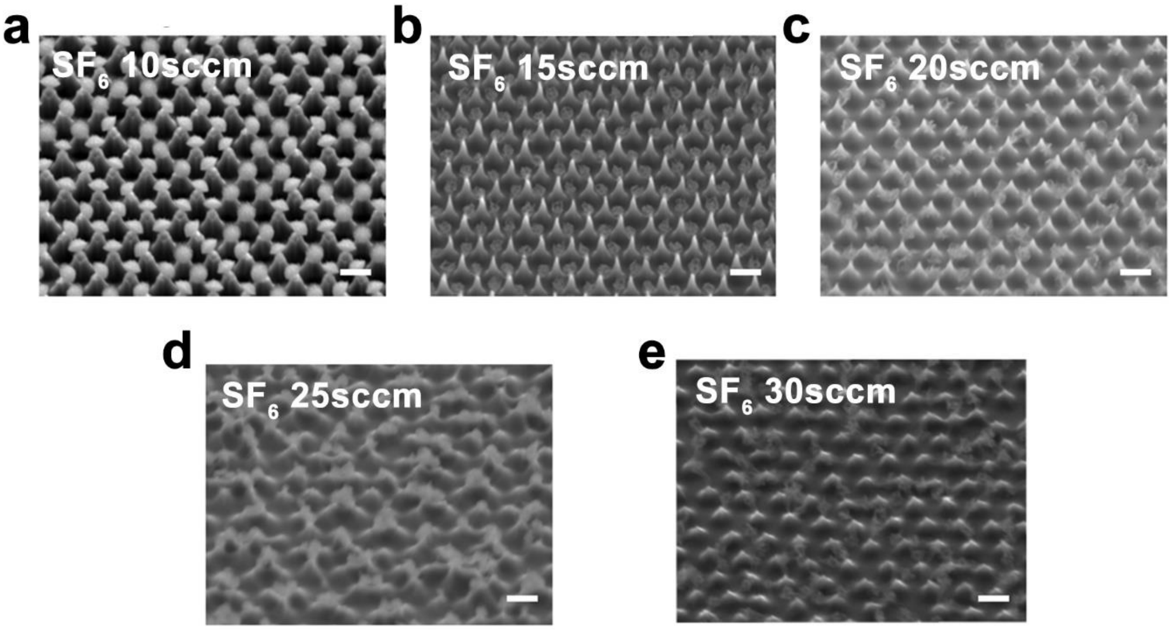

Figure S4. Effect of SF_6 flow rate on nanoneedle morphology during one-step ICP etching. SEM images of nanostructures fabricated using 1 μm PS microsphere masks at SF_6 flow rates of **(a)** 10, **(b)** 15, **(c)** 20, **(d)** 25, and **(e)** 30 sccm. The O_2 flow rate and etching time were fixed at 20 sccm and 180 s, respectively. Increasing the SF_6 flow rate initially promoted silicon removal and nanoneedle formation, whereas excessive SF_6 enhanced lateral erosion and produced over-etched or blunted structures. Scale bars: 1 μm.

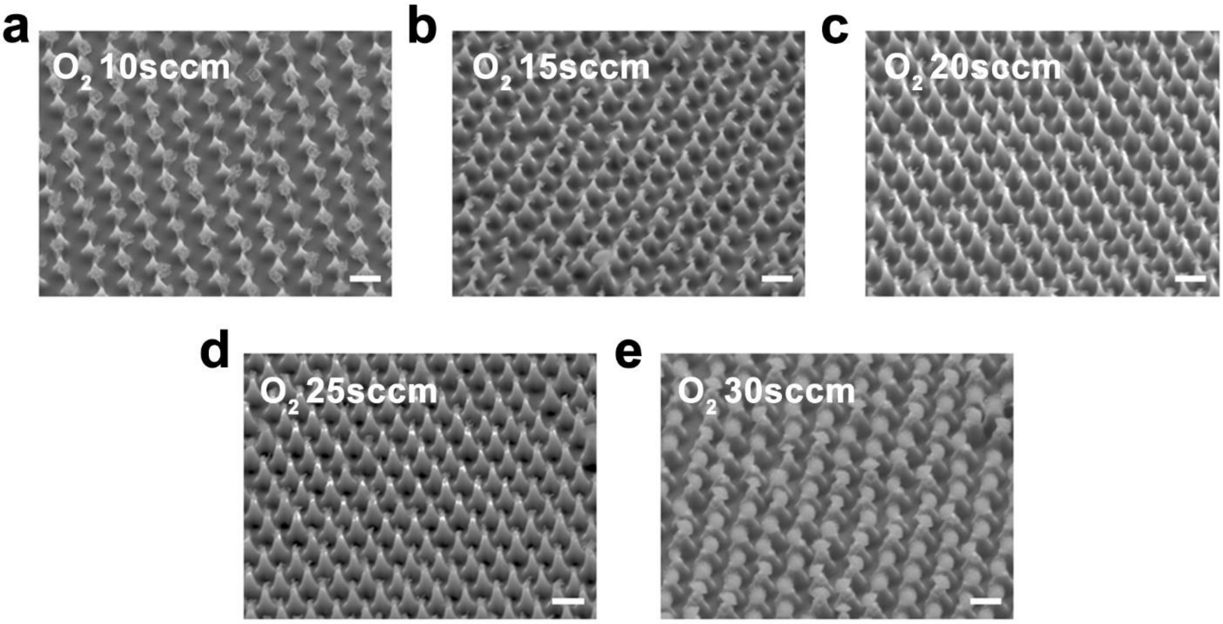

Figure S5. Effect of O_2 flow rate on nanoneedle morphology during one-step ICP etching. SEM images of nanostructures fabricated using 1 μm PS microsphere masks at O_2 flow rates of **(a)** 10, **(b)** 15, **(c)** 20, **(d)** 25, and **(e)** 30 sccm. The SF_6 flow rate and etching time were fixed at 15 sccm and 180 s, respectively. Insufficient O_2 provided inadequate sidewall protection and promoted lateral erosion, whereas an intermediate O_2 flow produced well-defined conical nanoneedles. Excessive O_2 suppressed vertical silicon removal and resulted in broader or under-etched structures. Scale bars: 1 μm.

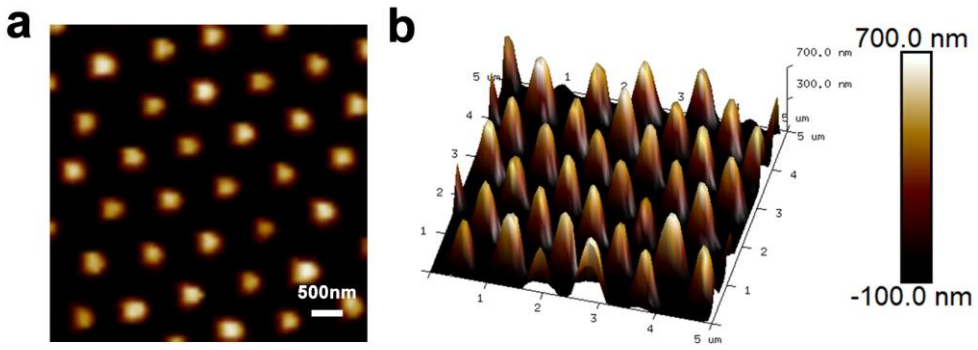

Figure S6. Tapping-mode AFM characterization of the one-step-etched nanoneedle array with 1 µm spacing. (a) Two-dimensional height image. Scale bar: 500 nm. **(b)** Three-dimensional representation of the surface topography. Color scale: -100 to 700 nm. The apparent lateral broadening and reduced topographic depth arise from finite-tip convolution and restricted probe access between adjacent nanoneedles.

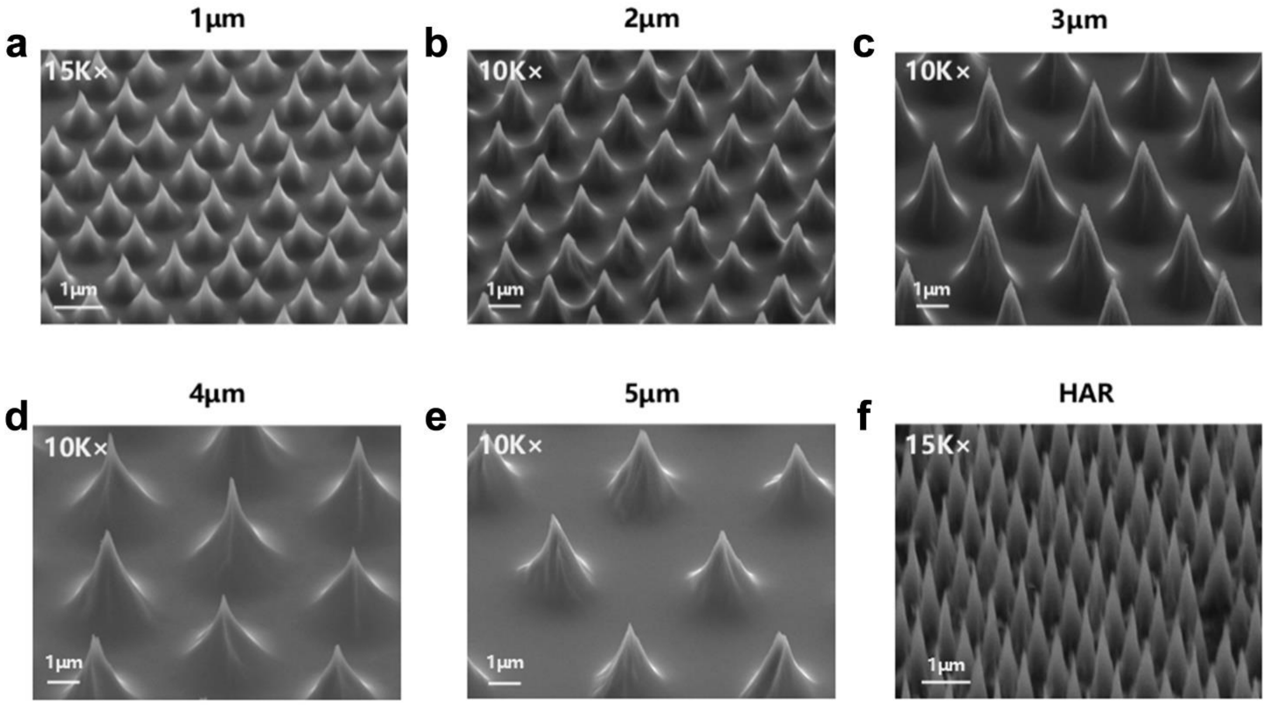

Figure S7. High-magnification tilted-view SEM images of the fabricated silicon nanoneedle arrays. (a–e) One-step-etched arrays with nominal center-to-center spacings of 1, 2, 3, 4, and 5 μm, respectively. **(f)** HAR nanoneedle array with a nominal spacing of 1 μm. Scale bars: 1 μm.

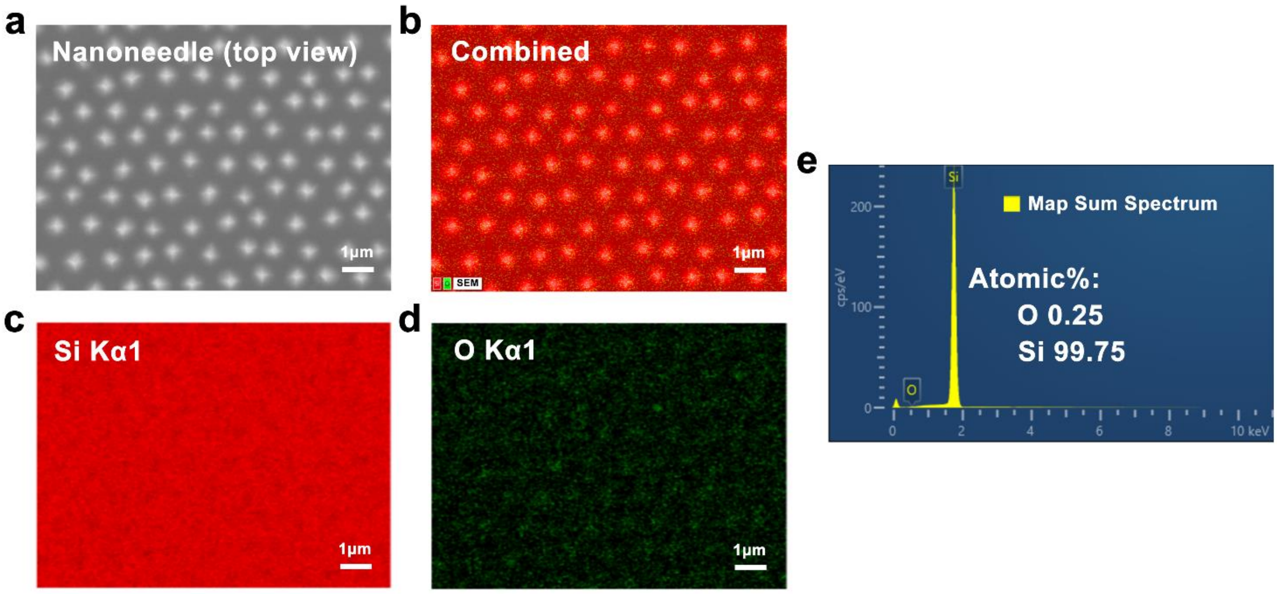

Figure S8. Elemental characterization of a representative one-step-etched silicon nanoneedle array with 1 μm spacing by energy-dispersive X-ray spectroscopy (EDS). (a) Top-view SEM image of the uncoated nanoneedle array. **(b)** Combined EDS elemental map of Si and O. **(c,d)** Individual Si Kα_1 and O Kα_1 maps, respectively. **(e)** EDS map sum spectrum acquired over the mapped area. Semi-quantitative analysis of the map sum spectrum yielded atomic fractions of 99.75 at.% Si and 0.25 at.% O. Scale bars in **(a–d)**: 1 μm.

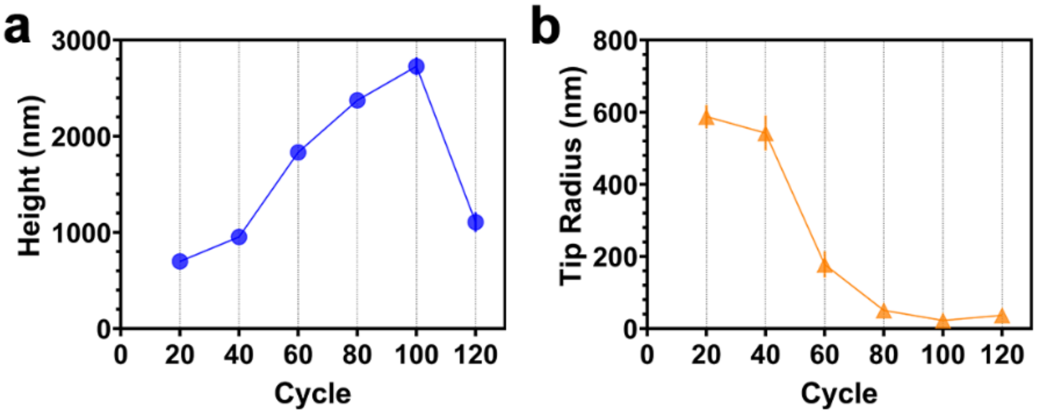

Figure S9. Evolution of HAR nanoneedle morphology with pseudo-Bosch etching cycles. (a) Statistics of nanoneedle height with different etching cycles. **(b)** Statistics of nanoneedle tip radius with different etching cycles. Data are presented as mean ± SD.

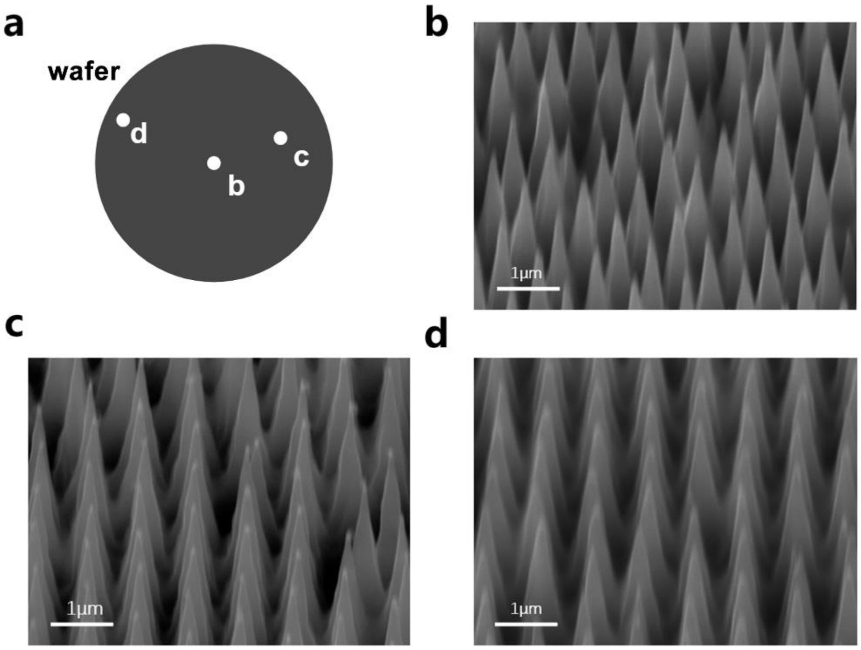

Figure S10. Spatial morphology of the high-aspect-ratio silicon nanoneedle array at three sampling locations. (a) Schematic showing three spatially separated sampling locations on the fabricated sample. **(b–d)** Representative tilted-view SEM images acquired at the corresponding locations indicated in **(a)**, showing similar nanoneedle morphology within the same sample. Scale bars: 1 μm.

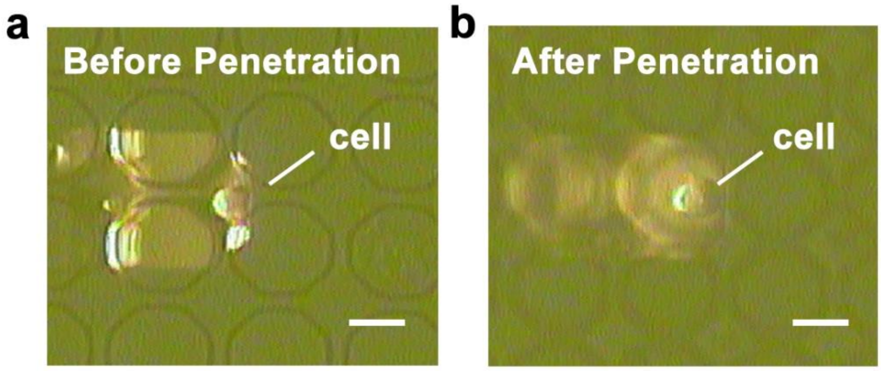

Figure S11. Representative bright-field images of the same cantilever-bound Jurkat E6-1 cell. (a) before force measurement. **(b)** immediately after force measurement. Scale bars: 20 μm.

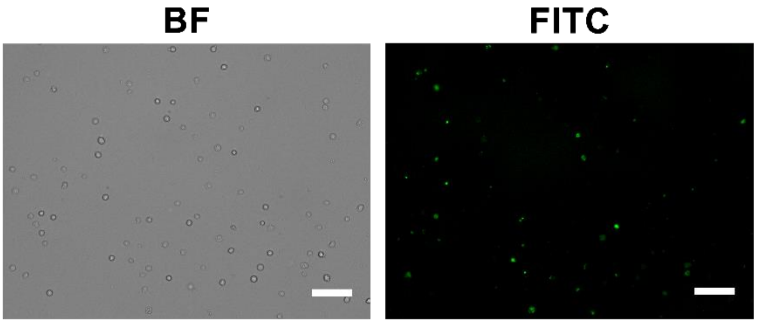

Figure S12. Fluorescence observation of Jurkat E6-1 cells after 48 h of post-delivery culture. Representative bright-field (left) and FITC fluorescence (right) images of cells after intracellular delivery using one-step-etched nanoneedle arrays with 4 μm spacing. After delivery, the collected cells were resuspended in complete RPMI-1640 medium and cultured at 37 °C for 48 h before imaging. Scale bars: 100 μm.

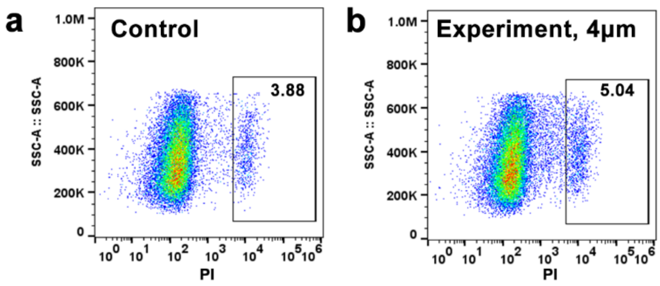

Figure S13. Flow-cytometry plots of propidium iodide (PI)-stained Jurkat E6-1 cells after delivery experiments. The PI-positive fractions in the Control and Experiment groups were 3.88% and 5.04%, respectively.

Statistics of first penetration event forces						
**Nanoneedle array**	**Spacing (μm)**	**Cell Number (*N*)**	**Mean ± SD (nN)**	**SEM (nN)**	**Median [IQR] (nN)**	**Min–max (nN)**
HAR	1	27	25.90 ± 12.98	2.50	21.31 [15.89–30.70]	9.75–58.58
One-step-etching	1	21	34.87 ± 13.29	2.90	37.12 [24.61–42.59]	12.01–56.00
One-step-etching	2	23	16.68 ± 5.96	1.24	17.21 [12.00–21.49]	4.51–26.68
One-step-etching	3	26	10.41 ± 3.97	0.78	9.88 [7.81–11.65]	5.00–19.30
One-step-etching	4	15	7.25 ± 6.15	1.59	4.04 [3.25–8.95]	2.43–22.00
One-step-etching	5	11	4.05 ± 0.99	0.30	3.85 [3.43–4.85]	2.37–5.64

Table S1. Descriptive statistics of cell-level first membrane-penetration forces across different nanoneedle arrays. Cell-level F_0 was defined as the mean first penetration force of all penetration-positive curves within each 16-curve cell map. SD, standard deviation; SEM, standard error of the mean; IQR, interquartile range.

Research question	Does F_0 decrease monotonically with increasing nanoneedle spacing?	Does HAR morphology reduce F_0 compared with one-step etching 1 um nanoneedles?	Does HAR increase penetration probability compared with one-step etching 1 um nanoneedles?
Varaible	F_0	F_0	Penetration probability
Dataset	One-step etching, 1–5 μm	HAR versus one-step etching 1 μm	HAR versus one-step etching 1 μm
N	96	27(HAR) versus 21(1μm)	28(HAR) versus 23(1μm)
Test	Spearman rank correlation	Mann–Whitney U test	Mann–Whitney U test
Statistics	ρ = −0.825	U = 175; rank-biserial r = −0.383	U = 445; rank-biserial r = 0.382
p value	4.94×10^{-25}	0.0248	0.0194

Table S2. Statistical analysis of spacing- and morphology-dependent membrane penetration. Spearman rank correlation was used to evaluate the monotonic association between nanoneedle spacing and cell-level first penetration force, F_0, across one-step-etched arrays with spacings of 1–5 μm. Matched-spacing comparisons between HAR and one-step-etched 1 μm arrays were performed using two-sided Mann–Whitney U tests to evaluate morphology-associated differences in cell-level F_0 and penetration probability. Cell-level F_0 was calculated as the mean first penetration force of all penetration-positive curves within each 16-curve cell map; cells without detected penetration events had no defined F_0 and were excluded from the F_0 analyses. Penetration probability was calculated as the number of penetration-positive curves divided by 16 for each cell, with zero-penetration cells retained. The Spearman correlation coefficient, ρ, and rank-biserial correlation, r, are reported as effect-size measures. HAR, high-aspect-ratio nanoneedle array.

Supplementary Note S1. Geometric basis of the nanoneedle array density-scaling model

The one-step-etched nanoneedle arrays were generated from close-packed PS microsphere monolayers and were therefore approximated as two-dimensional hexagonal lattices. For a hexagonal lattice with a center-to-center spacing *p*, the area associated with each nanoneedle is:

$$A_{cell} = \frac{\sqrt{3}}{2} p^2$$

Therefore, the density of nanoneedles could be described as:

$$\rho = \frac{1}{A_{cell}} = \frac{2}{\sqrt{3}p^2}$$

If the effective cell–array contact area immediately before the first penetration event is A_{eff}, and a fraction η of the nanoneedles within this region effectively participates in load sharing, the number of engaged nanoneedles can be approximated as:

$$N_{eff} = \eta A_{eff} \rho = \frac{2\eta A_{eff}}{\sqrt{3}p^2}$$

Assuming a critical penetration threshold f_c for each nanoneedle, the apparent force at the first penetration event can be expressed as:

$$F_0 = F_{inf} + N_{eff} f_c = F_{inf} + \frac{K}{p^2}$$

Where

$$K = \frac{2\eta A_{eff} f_c}{\sqrt{3}p^2}$$

Here, F_{inf} is an empirical spacing-independent offset that approximates the penetration force in the large-spacing limit, where the cell is assumed to engage effectively with a single nanoneedle. K is a composite density-scaling coefficient. However, K should not be interpreted as a single-nanoneedle material constant because it incorporates the effective contact area, the fraction of engaged nanoneedles, and the local penetration threshold. The p^{-2} term arises directly from the density scaling of a hexagonal nanoneedle lattice, which assumes that A_{eff}, η, and f_c remain approximately constant across the spacing series. In practice, spacing-dependent changes in cell deformation and membrane curvature may contribute to deviations between the experimental measurements and model predictions. Thus, the model should be interpreted as a first-order phenomenological approximation rather than a complete mechanical law of cell–nanoneedle penetration.
